# Recommendations for Standards of Network Care for Patients with Parkinson’s Disease in Germany

**DOI:** 10.3390/jcm9051455

**Published:** 2020-05-13

**Authors:** Tino Prell, Frank Siebecker, Michael Lorrain, Carsten Eggers, Stefan Lorenzl, Jochen Klucken, Tobias Warnecke, Carsten Buhmann, Lars Tönges, Reinhard Ehret, Ingmar Wellach, Martin Wolz

**Affiliations:** 1 Department of Neurology, Jena University Hospital, 07740 Jena, Germany; 2 Center for Healthy Ageing, Jena University Hospital, 07740 Jena, Germany; 3 Praxis Neurologie, 48291 Telgte, Germany; fs@neurologie-telgte.de; 4 Nervenarztpraxis Gerresheim-Pempelfort, 40477 Düsseldorf, Germany; dr.lorrain@volggerconsult.de; 5 Department of Neurology, University Hospital Marburg, 35037 Marburg, Germany; Carsten.Eggers@uk-gm.de; 6 Professorship for Palliative Care, Paracelsus Medical University, 5020 Salzburg, Austria; stefan.lorenzl@pmu.ac.at; 7 Department of Palliative Medicine, Ludwig-Maximilians-University Munich, 81377 Munich, Germany; 8 Department of Neurology, Klinikum Agatharied, 83734 Hausham, Germany; 9 Department of Molecular Neurology, University Hospital Erlangen, Friedrich-Alexander University Erlangen-Nürnberg (FAU), 91054 Erlangen, Germany; Jochen.Klucken@uk-erlangen.de; 10 Medical Valley-Digital Health Application Center GmbH, 96047 Bamberg, Germany; 11 Fraunhofer Institute for Integrated Circuits, 91058 Erlangen, Germany; 12 Department of Neurology, University of Muenster, 48149 Münster, Germany; Tobias.Warnecke@ukmuenster.de; 13 Department of Neurology, University Medical Center Hamburg-Eppendorf, 20246 Hamburg, Germany; buhmann@uke.uni-hamburg.de; 14 Department of Neurology, St. Josef-Hospital, Ruhr-University Bochum, 44801 Bochum, Germany; lars.toenges@rub.de; 15 Praxis Neurologie, 12163 Berlin, Germany; dr.ehret@neurologie-berlin.de; 16 Office for Neurology and Psychiatry Hamburg Walddörfer, Wiesenkamp 22 c, 22359 Hamburg, Germany; ingmar.wellach@immanuelalbertinen.de; 17 Department of Neurology, Ev. Amalie, Sieveking Hospital, 22359 Hamburg, Germany; 18 Department of Neurology, Elblandklinikum Meißen, 01662 Meißen, Germany; Martin.Wolz@elblandkliniken.de

**Keywords:** networks, telemedicine, multimodal complex treatment, day clinic, advanced care planning

## Abstract

Although our understanding of Parkinson’s disease (PD) has improved and effective treatments are available, caring for people with PD remains a challenge. The large heterogeneity in terms of motor symptoms, nonmotor symptoms, and disease progression makes tailored individual therapy and individual timing of treatment necessary. On the other hand, only limited resources are available for a growing number of patients, and the high quality of treatment cannot be guaranteed across the board. At this point, networks can help to make better use of resources and improve care. The working group PD Networks and Integrated Care, part of the German Parkinson Society, is entrusted to convene clinicians, therapists, nurses, researchers, and patients to promote the development of PD networks. This article summarizes the work carried out by the working group PD Networks and Integrated Care in the development of standards of network care for patients with PD in Germany.

## 1. Introduction

Parkinson’s disease (PD) is the second most common neurodegenerative disorder (after Alzheimer’s disease) and is predicted to increase in prevalence as the population ages [1]. Classically, PD is characterized by motor symptoms, namely, tremors, rigidity, and bradykinesia. However, the clinical phenotype of PD is highly variable, and a wide range of nonmotor symptoms (NMSs) underpins the clinical stages of PD. These NMSs, such as neurobehavioral disorders, cognitive impairment, and gastrointestinal dysfunction, are, by all accounts, very common in individuals with PD and contribute to poor quality of life [2]. The appearance of motor symptoms and NMSs vary throughout the disease course, which provides a challenge for care management not only for the neurologist but also for the general practitioner (GP), who is a frequent care contact for the patient. There are many pharmacologic therapies currently available, such as tablets, patches, pens, and pumps; deep brain stimulation (DBS) as an established surgical intervention; magnetic resonance (MR)-guided focused ultrasound as a new innovative procedure; and nonpharmacological interventions such as physiotherapy, speech therapy, and occupational therapy.

Importantly, the number of patients that are hospitalized for treatment of PD is constantly rising in Germany, and there are substantial regional differences in the availability of specialized treatment [3,4]. Definitively, clinical heterogeneity, as well as the availability of a wide variety of specialized therapeutic procedures, makes multiprofessional care necessary. PD requires close interactions between different care partners, for example, GPs, neurologists, therapists, nursing staff, social workers, and PD nurse specialists. However, the reality of treatment is that there are often significant gaps in care [5]. Proven structural concepts for the care of patients with PD are currently not well established in Germany. For example, it is not specified who should make the diagnosis of PD, who initiates and continues treatment and who continuously ensures guideline-adapted procedures [6]. In particular, the planning and implementation of therapy options for advanced PD reach their limits without an optimal setting. In Germany, people with PD are treated in general neurological practices, specialist medical practices, outpatient clinics, medical care centers, general university outpatient departments, and specialized university outpatient departments, as well as in GP practices. Insufficient communication between the various players can lead to different approaches to diagnostics and therapy and ultimately decrease patient adherence. In addition, patients increasingly request a so-called second or third opinion in specialized PD centers.

In recent years, it has been recognized that the formation of networks can contribute to improving the care of patients with PD. The establishment of networks for the treatment of patients with PD is a great challenge due to inhomogeneous regional conditions, the lack of a suitable technical infrastructure, and the strict regulation and separation of the individual sectors in the German health care system [7]. PD networks generally aim to improve the care of patients with PD at various levels by involving different actors in the inpatient and outpatient sectors, thereby additionally reducing costs [8]. The most important components of PD networks are, among others, the bundling of expertise of different, specially trained professionals (professional empowerment), a strong and active involvement of patients in the care process (patient empowerment), and the networking of professionals with special expertise in the field of PD (team empowerment) [9]. In Germany, different approaches and methods for treating patients with PD have been established as a result of different regional characteristics. Networks can help to make diagnostics more accurate, faster, and more effective, while avoiding duplicate examinations [7]. In addition to traditional outpatient, semi-inpatient, and inpatient treatment, practice visits and telemedical methods can be used to optimize therapy in networks [10,11]. Several factors are crucial for a successful and functioning network: a specialized team, the application of evidence-based treatment guidelines, the selection of motivated practitioners, regular training, commitment to compliance with the guidelines, patient-centered treatment, and transparent outcome quality [9,12]. The core element of patient care within a network should be the implementation of a standardized treatment pathway. This defines the best possible sequence of treatment steps on the basis of guidelines and medical expertise. If possible, all patients within a network should be treated with specialized neurologists, registered neurologists, and GPs working in a collaborative manner. There should be flowing boundaries to allow equal, individual care concepts based on medical necessity. In our opinion, the establishment of such a treatment pathway must be individually oriented in each network to the corresponding network structures, regional characteristics, and resources available in each case, making simple transferability between different networks impossible. Nevertheless, core elements will certainly be available in different networks. This review, therefore, provides an overview of evidence-based recommendations for the network care of patients with PD within the framework of a multisectoral, multiprofessional setting. 

## 2. Roundtable Participants and Format

The authors met in Cologne in 2019 for a roundtable discussion and to organize the foundation of the working group PD Networks and Integrated Care, part of the Deutsche Gesellschaft für Parkinson und Bewegungsstörungen (DPG). The working group has the following aims: synchronization of supply networks in Germany, development of minimum standards, development of joint research projects, further development of nursing staff qualifications, and development of qualification standards for therapy groups. The DPG working group pursues the goal of improving patient care in close cooperation with other physicians, therapists, and patient support groups. The roundtable discussion was sponsored by DPG (travel costs). Following introductions and stated aims, various points of interest (existing German network structures and aims, communication strategies, standards of network care, etc.) were discussed. No formal votes were taken at the meeting. The discussions identified general points of agreement. 

## 3. The Patient Journey—Where to Go When PD Is Suspected?

To give recommendations for a standard of network care, one has to acknowledge the existing care paths for patients with PD in Germany. Typically, the initial symptoms are not identified as Parkinsonism or PD-specific symptoms by the patients themselves. Instead, they usually approach their GP with motor or even more important and frequent nonmotor complaints (e.g., obstipation, pain and depression). Occasionally, physiotherapists treating back pain or degenerative joint symptoms realize that these are the first signs of motor symptoms (e.g., rigidity) related to PD and that the patient should be referred for a PD diagnostic workup.

Patients who live in rural areas are more likely to have their GP identify symptoms as being related to PD (13). However, the GP model in Germany is not as rigid as in other countries, and many citizens not only have a primary care physician but can also consult specialists (e.g., internal medicine, orthopaedists, etc.), depending on their prior health care contacts and requirements throughout their lifetime.

## 4. What Symptoms and Complaints Should Prompt a GP to Consider a Diagnosis of PD, and When Should a Neurologist Be Consulted?

Parkinsonism refers to a clinical presentation characterized by the presence of bradykinesia plus rest tremor or rigidity [13]. Bradykinesia is a generalized slowing of movements and repetitive motion fatigue. It may present as hypomimia (‘‘masked face’’), hypophonia, worsening of fine-motor tasks, micrographia, difficulty turning in bed, or reduced arm swing with side difference. Additionally, distinct changes of gait and balance, like short steps, shuffling gait, and uncertainty when turning around are common. Rigidity is the resistance that can be assessed clinically by passively flexing and extending a patient’s limb. Typically, the patient complains about stiffness and pain, which often manifests as shoulder or back pain. While kinetic and postural tremors may occur, the rest tremor is the most common type of tremor in early PD. Patients with these clinical signs should be referred to a neurologist for further diagnosis, because, in Germany, the time between the appearance of the first symptoms and the diagnosis is significantly longer when patients with these symptoms see their GP [5]. Patients with symptoms that may be related to PD should be asked about PD-typical nonmotor signs such as sensory symptoms (loss of smell, pain), depressed mood, rapid eye movement–sleep behavior disturbance, periodic limb movement disorder, or constipation, which frequently occur years before motor signs are realized.

Thus, one recommendation for standard of care in the initial phase of the disease course is physician awareness of the first signs of PD (which could be achieved with better information and secondary prevention standards in the network) and early referral of patients to a movement disorder specialist (which could be achieved by specific disease management programmes). The movement disorder specialist should be a neurologist with many years of experience caring for patients with PD. In Germany, there are specific recommendations for patient referral in this context that can guide decisions in the outpatient setting [6].

## 5. What Diagnostic Tests Should Be Performed by the Neurologist at the First Visit?

In 2015, the official International Parkinson and Movement Disorder Society (MDS) Clinical Diagnostic Criteria for PD were proposed [13]. The benchmark for these criteria is an expert clinical diagnosis. However, the criteria can be easily applied by clinicians with less expertise in PD diagnosis [14].

In the MDS criteria, motor symptoms remain the core feature of the disease, defined as bradykinesia plus rest tremor or rigidity (explicit instructions for defining these symptoms are given). After consideration of absolute exclusion criteria (which rule out PD), red flags, and supportive criteria, the diagnosis of clinically established PD or probable PD can be made, or PD can be ruled out.

Besides anamnesis, clinical assessment with cerebral imaging (cranial MR imaging [MRI] is preferred) should be performed to exclude symptomatic causes of PD symptoms. In case of divergent clinical and MRI-based diagnoses, the clinical assessment should take precedence. However, a differentiated approach is required for the numerous subsequent apparatus and drug tests. The evidence shows that levodopa and apomorphine tests are not as meaningful as standard levodopa therapy in differentiating between established PD and atypical Parkinson’s syndrome. A negative test does not rule out a response to longer-lasting levodopa treatment. This suggests that levodopa and apomorphine tests should not be routinely used in differential diagnosis but may be valuable in specific clinical situations [15]. A reduction in olfactory capacity is a sensitive but not specific indicator of PD. Therefore, standardized olfactory tests are only recommended in combination with other diagnostic procedures for the diagnosis of PD. Striatal dopamine active transporter-single photon emission computed tomography (SPECT) imaging should be used early in the course of the disease to detect a nigrostriatal deficit in clinically unexplained parkinsonism or tremor syndrome [15]. In contrast, the postsynaptic (I 123-iodobenzamide) SPECT should not be used for the differential diagnosis of established Parkinson’s syndrome (syn. Idiopathic Parkinson Syndrome) to differentiate atypical neurodegenerative disease variants. The myocardial 123 MIBG-SPECT can be used to distinguish multiple system atrophy from PD [15]. In addition, functional brain imaging with positron emission tomography is a valuable diagnostic tool for the differential diagnosis of idiopathic Parkinson Syndrom and atypical parkinsonism [16,17].

For clinical neurological confirmation of the diagnosis and therapy control, the patient should be examined after 3 months, and thereafter according to clinical need but at least once a year [15]. Because even proven experts have to revise the diagnosis of an IPS during the course of the disease, the diagnosis should be reviewed at regular intervals. With the rising availability of electronic patient records, another recommendation is that a standard set of information should be generated and stored in the record of each patient with PD after the results of the first diagnostic tests. This information should be available for the patient and his or her health care provider team.

## 6. Which Patients Should Be Referred to a Specialized Movement Disorder Center?

Referral to a movement disorder specialist is important to improve the accuracy of diagnosis, for case selection and to provide guidance in terms of specialized device-aided therapies, namely, DBS, levodopa/carbidopa intestinal gel (LCIG) and apomorphine. Consultation from the medical staff of a specialized center may improve motor function and the quality of life in patients in advanced PD stages [18,19]. Patients with the following constellations and symptoms should be referred to a movement disorder specialist even if the disease duration is <4 years [20,21,22]:

- Doubts about correct diagnosis;
- Young onset and side effects under prescribed medication;
- Troublesome motor fluctuations;
- ≥1 h of troublesome dyskinesia/day;
- ≥2 h ‘off’ symptoms/day;
- ≥5 oral levodopa doses/day;
- Freezing of gait;
- Acute or subacute camptocormia;
- High burden of NMS;
- Nontransitory troublesome hallucinations;
- Repeated falls despite assumed treatment.

Referral to a movement disorder specialist should also be considered for the patient to have access to the most innovative treatment and clinical research options. A substantial number of patients are highly interested in contributing to research, the opportunities for which are typically limited to regional neurologists. Recommendations for these patient referrals in the outpatient setting in Germany have been proposed [6].

## 7. Which Patients Should Be Admitted for PD Multimodal Complex Treatment (MCT)?

Some health care insurance systems reimburse treatment of patients with PD in specialized units. A well established and frequently used multiprofessional inpatient treatment concept in Germany is PD multimodal complex treatment (PD-MCT). Prerequisites for patients taking part in MCT are documented physician diagnosis of PD, a constant anti-Parkinsonian drug titration, and the application of activating therapies (at least 7.5 h/week). It involves physicians, physiotherapists, occupational therapists, speech therapists, and other specialists for the optimization of PD treatment [3] and usually lasts 7 to 21 days. This therapy programme has been shown to be effective, with a reduction of motor symptoms and NMS [23,24]. Richter et al. [3] performed an analysis of 55,141 inpatients with PD who were integrated into this MCT from 2010–2016. They found that a large majority of patients with PD need to leave their residence county for an inpatient stay in a specialized PD unit. This limited access to multimodal therapy programmes means that patients sometimes have to travel long distances to receive specialized therapy [3]. There are no generally valid definitions of which patients should be treated within the complex programme and which should not. In view of the heterogeneity, it is difficult to make binding statements about this.

A prerequisite should be that the motor or NMSs can no longer be satisfactorily treated by outpatient therapy. Another prerequisite should be that patients are dealing with limitations in their activities of daily life and have a reduced quality of life. This can be the case, for example, with side effects under oral therapy, motor deterioration, or the high burden of NMS. Other typical indications for inpatient treatment would be the discontinuation of DBS or the initiation and optimization of therapy with LCIG or apomorphine. However, as the disease progresses and progressive limitations in mobility and cognition are observed, the benefits of inpatient treatment must be weighed against the increasing risk of delirium.

Overall, clinical experience shows a substantial benefit of PD-MCT for a large number of patients. The preselection process could ideally be managed by network structures and players. Additionally, the positive effect achieved by intense medical and nonmedical intervention should be maintained after release by immediate intensified ambulatory intervention and home-training concepts in order for the patients to benefit from the positive experience. This would be an important incentive for the patient to take part in PD-MCT.

## 8. Which Patients Should Be Admitted to a PD Day Clinic?

For patients with PD who need to adapt to complex medication schemes, drug pumps, or DBS devices, a classical outpatient or inpatient setting is not appropriate to sufficiently address clinical problems, while in a neurologist’s office or even in a movement center, outpatient clinic time and staff capacities are limited and the results of changes in medication or stimulation of the DBS device can only be monitored in the next (often late) consultation. An in-house stay is associated with an artificial environment that does not reflect the individual’s everyday life demands and is less suited for patients with dementia who often cannot cope with an altered environment. Furthermore, many patients with PD decline hospitalization for personal reasons such as job issues or having to care for other family members. For these patients, at the border between inpatient and outpatient care and the need for sophisticated treatment strategies, the new comprehensive, individual, and interdisciplinary concept of a PD day clinic has proven to be effective [25]. In the meantime, in Germany, several university clinics with a PD focus have established this or a similar PD day clinic concept to close the gap in PD care that have been found to be a transnational issue [26,27,28]. The concepts and standards of qualified PD day clinics have been certified recently by the TÜV and the German Parkinson Patient Society [29].

## 9. Who Else Should Be Involved in the Treatment of Patients with PD?

In general, a neurologist should be responsible for long-term medical care of patients with PD, and movement disorder specialists should be involved when there is a special issue. However, for various reasons, this is not always possible. Neurologists may not be available in rural areas, and even for patients in nursing homes, access to specialized neurological treatment is often limited. This is an important issue, because the number of patients in long-term care facilities will rise sharply in the coming decades [30]. For patients with PD, the interaction between the GPs and neurologists is essential. PD networks can make a decisive contribution to ensuring high-quality care of these patient groups.

Medical treatment is not the only option to control the motor symptoms and NMS during the course of the disease. Other nonmedical treatment options from other specialists are frequently necessary to improve functional status, performance of daily activities, and quality of life. These specialists include, among others, physiotherapists, occupational therapists, speech therapists, PD nurse specialists, and social workers [31]. Specific recommendations for physiotherapists, physicians, and patients with PD were published in the European Physiotherapy Guidelines for Parkinson’s Disease [32].

Health professionals must have sufficient PD-specific knowledge and expertise [33]. Physiotherapy has a positive impact on functional activities involving gait, transfers, and balance [32,34]. The occupational therapist focuses on enabling performance and engagement in meaningful activities [35]. Home-based, individualized occupational therapy can improve the self-perceived performance of daily activities in patients with PD [36]. Timely referral to physiotherapy, and occupational therapy is recommended because difficulties in daily activities can occur in every disease stage. Given the high prevalence of dysphagia and dysarthria during the course of the disease [37], speech-language therapy, including swallowing techniques, is frequently necessary for patients with PD. A collaborative approach between these disciplines should focus on complementary and different aspects. Therapists have to be aware of each other’s expertise, and effective and timely communication is essential [35]. PD networks are promising tools to share information about diagnostic results, current treatment goals, and plans.

In addition, there are many different nonphysician PD specialists for inpatient and outpatient care, such as PD nurse specialists or Parkinson assistants (PASSs). Their different roles and functions are described in another paper in this Issue. Depending on the location (inpatient or outpatient), the focus of their tasks can be different. These specialists are often familiar with aspects of case management; medication adherence; provision of information, education, psychosocial support, and coping skills; and caregiver support [38]. Patients with PD should have 1) regular access to clinical monitoring and adjustment of medication in consultation with the treating physician; 2) regular contact with caregivers, including home visits, as appropriate; and 3) access to reliable sources of information on clinical and social issues affecting patients with PD and their caregivers/families. These functions could be provided by PD nurse specialists or a PASS. The positive therapeutical effects of PD nurse specialists are currently evaluated for their health economic impact [39].

In particular, patients with advanced PD may benefit early from palliative care. Doctors and nursing staff can provide information about the final phase so that the family can take advantage of adequate care options. Palliative care should be aligned with patient priorities and complement other treatments. Therefore, advanced care planning might also increase knowledge about end of life issues. Generally, it should start early in the course of the disease. It can be started when particular symptoms occur (pain, dyspnoea, dysphagia, and aspiration) or at the very end of life [40,41]. Besides general markers of advanced disease (frequent infections and hospitalizations, malnutrition, etc.), the palliative performance scale can be used to measure the functional status of a patient and to determine the eligibility for enrolment in a palliative care programme [41,42]. Dysphagia with symptomatic aspiration might be taken as a clear indicator when palliative care should begin, because it also involves a discussion about life-prolonging therapies such as tube feeding.

**Figure 1:**
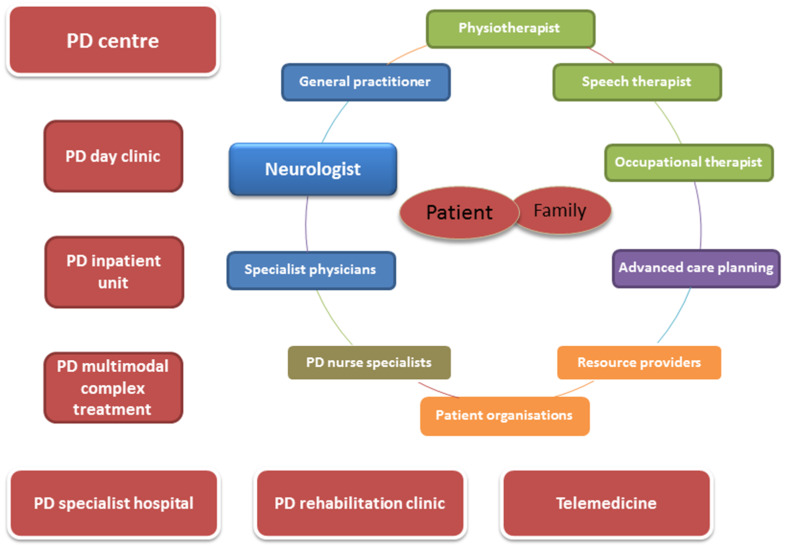
Schematized representation of common players and structures in a local supply network.

Figure 1 provides an overview of common players and structures in a local supply network.

The therapist network (outpatient) directly surrounding the patient and his or her environment is not only linked to the patient, but therapists are also linked to each other. This results in mutual inter-relationships and a flow of information between all professional groups involved (not only between the directly neighbouring ones). A supraregional supply network in the form of clinics and centers is connected to this ‘’micro-network’’. Here, exchange and cooperation results. Different stationary and semistationary care options are offered and supplemented with, for example, telemedical services (e.g., medical video observation and sensor-based motion analysis).

## 10. What Is the Relevance of Self-Management in Patients with PD?

Self-management means having knowledge, skills, and confidence to manage daily tasks when living with a chronic disorder such as PD. It includes the concepts of self-management tasks (medical, role, and emotional management) and self-management skills (problem solving, decision-making, resource utilization, the formation of a patient–provider partnership, action-planning, and self-tailoring) [43]. Patients with PD should be able to monitor progress and problems and to set, communicate, and harmonize their individual therapeutic goals with all members of the health care provider team. In addition, required information for the individual aspects of the disease symptoms, treatments, and side effects/risks should be tailored to the patient requirements and transferred adequately to the patient. Health care providers involved in the care of patients with PD can positively influence self-management skills with distinct approaches that mainly focus on education and support. Self-management in PD may, therefore, contribute to slower disease progression, reduced complications, and lowered costs [44]. However, self-management support interventions for patients with PD vary in content, structure, and intensity, and little is known about which existing self-management support programmes are most effective. As indicated by a recent overview of self-management support programmes for patients with PD, clinicians should ensure that the key components of education, goal setting, and guided problem solving are included. Moreover, adding these skills to the rehabilitation process and including caregivers and peer support systems seems promising [44].

## 11. What Is the Role of Telemedicine in the Network Care of Patients with PD?

As mentioned above, PD requires close interaction between different care partners in order to provide the best possible care for the patient. Rural location, nursing home residence, and the presence of physical or cognitive impairment are common reasons for limited access to specialized PD health care [45]. A PD network can improve access to specialized health care and manage the distribution of resources, tasks, and responsibilities. By doing so, PD networks can help to avoid unnecessary hospitalization and reduce costs [8]. Different methods exist to bring PD-specific knowledge and care to the patients in a PD network structure. In this context, telemedicine has shown promising effects for the management of PD. This includes synchronous methods (videoconferencing) and asynchronous methods (e.g., e-mail, smartphone assessments, remote monitoring, and wearable devices) [10,11,46]. Telemedicine has the potential to allow PD-specific efficient care to be delivered to more patients and more regularly than a traditional model of care [47]. From the patient’s view, telemedicine has the advantages of access to specialists, convenience, and time savings [48]. At present, it is applied in several clinical settings due to sanctions imposed for infection prophylaxis in the current sars-cov-2 pandemic, and it is seen to be a suitable tool with which to give advice and treat patients with PD. It also can be used to support outpatient palliative care teams with special neurological knowledge when the patient chooses to die at home [49]. Since 2019, the remuneration of video consultation hours has been based on the insured, basic, or consultation flat rate in Germany. Nevertheless, telemedicine is still limited by patients’ limited access to high-speed internet and usability issues (especially in elderly patients) [46]. Nevertheless, with the new Digital Health Act (‘’Digitale-Versorgung-Gesetz’’ (DVG)), reimbursement for video-based home telemedicine support has begun in Germany, and now, home telemedicine needs to be integrated into PD health care workflows.

## 12. Future Directions

The German health care system is struggling with the issues of separation of care sectors (e.g., outpatient vs. inpatient care) and considerable differences in the provision of care in urban and rural areas. In order to optimize the specialized care of patients with PD in Germany, the current care structures must be changed. This can be achieved by establishing PD networks, which act as a link between outpatient and inpatient treatment as well as between patients, caregivers, GPs, nonspecialized neurologists, movement disorder specialists, and other therapists. This is a promising way to ensure that a stage-appropriate and patient-specific therapy for PD can be initiated promptly and maintained permanently in accordance with the current guidelines. Additionally, new e-health processes might overcome current barriers and limited access to specialized health care and provide both patients and health care professionals with the potential for future seamless care, a strong interaction between health care partners, and involvement of patients and caregivers. Interestingly, many patients with PD are using digital media tools and smartphones and thus have access to digital technology [50]. Furthermore, the recently released Digital Health Act (DVG) will enable patient-centered technologies as digital health care applications for better support of trans-sectoral PD health care.

Especially against the background that some studies have found only limited benefits from specialized network structures, it is very important to provide scientific support for the formation of networks in Germany. These studies from England or the Netherlands that focus on very limited aspects (e.g., PD nurses, physiotherapy) are only transferable to Germany to a limited extent [33,51]. Decision makers, planners, and managers need evidence-based policy options and information on the scope of networks [52]. The DPG working group Networks and Integrated Care is therefore an opportunity to provide a framework for various forms of networks, to facilitate the exchange of experience, and to provide scientific support for the various structures and networks with their regional characteristics.
